# Vertebral carcinomatosis eleven years after advanced gastric cancer resection: A case report

**DOI:** 10.3892/ol.2014.2822

**Published:** 2014-12-23

**Authors:** FRANCESCO IOVINO, MICHELE ORDITURA, PASQUALE PIO AURIEMMA, FRANCESCA ROMANA CIORRA, GIOVANNI GIORDANO, CONSIGLIA ORABONA, FRANCESCO BARA, RENATO SERGIO, BEATRICE SAVASTANO, ALESSIO FABOZZI, MARIA MADDALENA LATERZA, JOLE VENTRIGLIA, ANGELICA PETRILLO, CARMINIA MARIA DELLA CORTE, FERDINANDO DE VITA

**Affiliations:** 1 IX Division of General Surgery, Department of Anaesthetic, Surgical and Emergency Sciences, Second University of Naples, Naples I-80131, Italy; 2 Division of Medical Oncology, ‘F. Magrassi - A. Lanzara’ Department of Clinical and Experimental Medicine, Second University of Naples School of Medicine, Naples I-80131, Italy

**Keywords:** vertebral metastases, gastric cancer, tumour dormancy

## Abstract

Bone metastasis is an uncommon event in advanced gastric cancer patients and bone metastases are rarely detected as isolated lesions. However, eleven years after treatment for locally advanced gastric cancer, including total gastrectomy followed by adjuvant chemotherapy, a 49-year-old female was admitted to the IX Division of General Surgery of the Second University of Naples (Naples, Italy) exhibiting severe progressive neurological symptoms. Magnetic resonance imaging indicated vertebral abnormalities, with evidence of marrow infiltration in several vertebral bodies; however, a contrast-enhanced computed tomography scan did not detect disease progression to other sites. Biopsy of the soft tissue at the level of the second lumbar vertebra (L2) revealed a metastatic lesion derived from gastric mucinous adenocarcinoma. The patient was initially treated with radiotherapy directed to the L2–L4 vertebral bodies to control the pain. Subsequently, systemic chemotherapy according to a FOLFOX-4 (leucovorin, fluorouracil and oxaliplatin) regimen commenced. However, after eight cycles, pulmonary progression of the disease occurred. Thus, palliative care was administered and the patient succumbed one month later. The late relapse of gastric cancer in the current patient may be associated with the theory of tumour dormancy.

## Introduction

Gastric cancer (GC) is the fourth most common malignancy, and the third leading cause of cancer-related mortality among males and the fifth leading cause of cancer-related mortality among females worldwide, with ~1,000,000 new patients diagnosed every year (1). Curative resection is the most successful treatment modality for locally confined GC (2), and the five-year relative survival rate is ~29% (3). Recurrence of GC generally occurs early, predominantly within the initial two years following gastrectomy. Late recurrence is uncommon and is extremely rare >10 years after gastrectomy (4). The majority of advanced GC patients eventually develop cachexia and peritoneal carcinomatosis, and succumb to multiple organ failure within five years (4). Advanced-stage cancer patients demonstrate a significantly higher incidence of bone cancer recurrence, and the majority develop combined recurrences, most commonly lymph node metastasis and peritoneal carcinomatosis (5). Solitary bone metastases are only observed in one-third of cases (6–10). In the present study, we report a rare case of relapse from advanced GC with extensive vertebral metastases and bone marrow infiltration at an 11-year follow-up. The theory of tumour dormancy may explain this phenomenon. Written informed consent for the publication of this study was obtained from the patient.

## Case report

### Clincial history

In September 2001, a 49-year-old female was admitted to the IX Division of General Surgery of the Second University of Naples (Naples, Italy) presenting with locally advanced GC. The patient underwent total gastrectomy with Billroth II anastomosis and D2 lymphadenectomy. The postoperative course was normal. Histological analysis identified an infiltrated mucinous adenocarcinoma with the presence of signet ring cells and metastases were identified in 2/32 dissected lymph nodes. Stage III cancer was diagnosed according to the fifth edition of the American Joint Committee on Cancer classification system (11) and the patient received an adjuvant chemotherapy treatment according to the ELFE regimen (60 mg/m^2^ epirubicin on day 1; 100 mg/m^2^ leucovorin and 375 mg/m^2^ fluorouracil on days 1–5; and 80 mg/m^2^ etoposide on days 1–3). This regimen was repeated every three weeks for six cycles (12). The patient was regularly followed-up at the outpatient clinic of our department every three months for the first two years and every six months until the fifth year. Furthermore, the patient underwent annual upper endoscopy, and chest and abdomen computed tomography (CT) scans. After five years, the patient was subjected to annual physical examinations, chest radiographs and abdominal ultrasound scans.

### Clinical presentation and diagnosis

**Figure 1:**
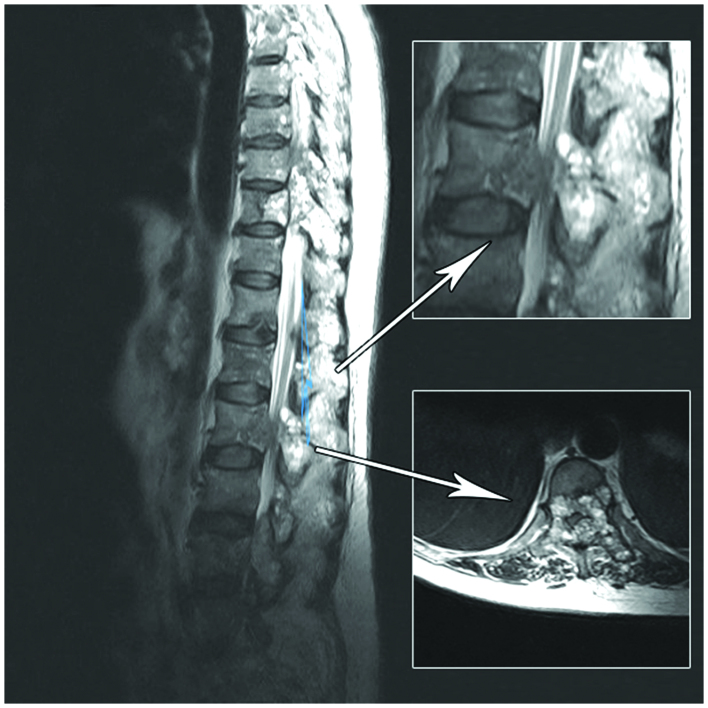
Magnetic resonance imaging of the vertebrae, demonstrating numerous abnormal vertebral bodies with marrow infiltration, particularly in the second lumbar vertebra. Upper and lower (inset) images indicate the sagittal and coronal planes, respectively.

In November 2012, the patient developed severe progressive paraparesis with the inability to maintain an upright posture, as well as retention of the sphincters. In addition, the patient complained of severe pain in the spine and left sciatica in the previous 2–3 weeks. Physical examinations conducted three months prior to the present symptoms did not reveal chest, abdominal or central nervous system indicators of relapse. Upon clinical examination, paraparesis with reduced tone and areflexia of the lower limbs was identified. Muscle power was determined to be 1/5 in the left and right legs. Magnetic resonance imaging (MRI) of the entire spine revealed numerous abnormal vertebrae; in particular, the thoracic (T4–T6, T9–T10), lumbar (L2–L4) and sacral (S2) vertebra. Furthermore, several vertebral bodies exhibited marrow infiltration, particularly L2 and L4 (Fig. 1), and a soft tissue mass was identified in the spinal cord canal from L2 to L4. The dural sac was displaced anteriorly with marked compression at the L2 level. Brain, chest, abdomen and pelvis CT scans were performed for tumour staging, which detected no signs of disease. A CT-guided biopsy of the soft tissue at the L2 level revealed metastatic adenocarcinoma with signet ring cells; specifically, the biopsy cells positively stained for mucin, cytokeratins 7 and 20, and carcinoembryonic antigen, whereas the cells were negatively stained oestrogen and progesterone receptors, as well as cancer antigen (CA) 125. These histological features were consistent with metastasis from gastric mucinous adenocarcinoma. Thus, human epidermal growth factor receptor 2 (HER2) expression levels were assessed in biopsy and resected gastric cancer tissue samples obtained from the patient in 2001; however, no HER2 overexpression was identified. The performance status was determined as two according to the Eastern Cooperative Oncology Group scale (http://www.ecog.org/general/perf_stat.html) and, with the exception of the bone marrow infiltration, no haematological disorders were detected.

### Treatment

Initial management was targeted to the control the patient’s severe back pain symptoms; thus, 20 Gy radiotherapy targeted to the L2–L4 vertebral bodies was performed. Subsequently, systemic chemotherapy according to FOLFOX-4 regimen commenced: 85 mg/m^2^ oxaliplatin; 200 mg/m^2^ leucovorin; and 400 mg/m^2^ fluorouracil (5-FU), intravenous bolus, followed by continuous infusion of 5-FU (2,400 mg/m^2^) over 46 h every two weeks (13). After eight cycles, a further progression of the disease occurred, with the patient exhibiting superimposed lung metastases. In consideration of the poor clinical condition of the patient, supportive care was administered. The patient succumbed one month later.

## Discussion

The first novel aspect of the present report is the uncommon location of the secondary lesions. GC usually metastasizes to the liver, peritoneum, lymph nodes and lungs, whereas bone metastases are uncommon, occurring in ~13.4% of autopsy cases in a Japanese study, and are rarely detected as isolated lesions (6–10). The metastatic path of neoplastic GC cells is generally hematogenous through the bone marrow, as the gastric mucosa has a rich capillary network and the bone marrow does not contain lymphatic vessels (14). This hypothesis is supported by the observation that a higher incidence of bone metastasis occurs in the axial skeleton, such as the spine, pelvic bones or the sternum, where there is a higher content of hematopoietic bone marrow in adults (15). Therefore, the bone marrow, rather than the bone tissue, is the target in bone metastasis.

The prognosis of patients exhibiting bone metastases from GC is worse compared with other solid tumours, with a mean survival period of <5 months and the longest survival period reported in the literature, 3.5 years (6). Early detection of bone metastases is difficult as, according to the majority of important international guidelines (16), skeletal examinations are only performed upon presentation of bony pain symptoms.

No standard treatment for bone metastases with marrow infiltration has been established and local approaches represent the most feasible therapeutic approaches. In the present report, in consideration of the multiple vertebral metastases, a palliative radio-chemotherapy approach was implemented. In the metastatic setting, treatment aims to control patient symptoms, improve patient quality of life and prolong patient survival; however, current chemotherapeutic approaches have limited efficacy and specific approaches exhibit unfavourable toxicity profiles. Fluoropyrimidine, taxanes and platinum-based regimens are the most commonly used chemotherapeutic approaches, providing response rates of 30–50% and a median overall survival of ~1 year (17). These data support the requirement for the development of novel therapeutic strategies based on targeted agents. Trastuzumab, a monoclonal antibody against HER2, has demonstrated a survival advantage when applied as a chemotherapeutic agent in various types of HER2-positive GC patients (13,18). However, the current patient was determined to be HER2-negative and, thus, did not benefit from the treatment.

The second novel aspect of the present report is the long disease-free period experienced by the patient. Relapse from GC usually occurs within five years after surgery and the median recurrence period is 28 months (range, 4–111 months) following surgery (2,5). Advanced cancer and poorly differentiated adenocarcinoma are associated with a high risk of relapse (6), hence, late recurrences are uncommon; <10% of patients recur after five years and <1% recur after 10 years (19). However, few studies of isolated bone recurrences ≥9 years after detection of the primary tumour have been reported (20). Recent studies on GC reported a 0.4–3.8% incidence of bone metastasis (20–23). The time elapsed between surgical resection and the onset of bone metastasis may be explained by the tumour dormancy theory, which proposes that a period of tumour progression exists in which the presence of tumour cells in distant organs does not increase the tumour burden. This condition is clinically translated as a long disease-free interval between primary tumour resection and relapse (21,22). Single dormant cells are defined as cells undergoing cycle arrest that have the ability to develop mechanisms to evade immune surveillance (24,25). Only a small proportion of dormant cells (~2%) develop into micrometastases, and an even smaller proportion (~0.02%) develop into macroscopic tumours (26). In micrometastatic dormancy, a state of balance exists between apoptosis and cell proliferation, resulting in no increase of tumour burden (27). Tumour dormancy ends after variable periods, up to years after the diagnosis of the primary tumour; subsequently, the cancer cells start to proliferate and late relapse occurs. The regulation of tumour dormancy entry and exit remains poorly understood, however, various factors may be involved, including genetic, and epigenetic changes, angiogenic switching, immune evasion and the microenvironment. The prevalence of clinical dormancy has been reported in numerous solid tumours, such as breast, renal, thyroid and prostate cancer, as well as melanoma, however, clinical dormancy has rarely been observed in GC (26).

In conclusion, vertebral metastases with bone marrow infiltration represent an uncommon occurence in GC, and their treatment can prove difficult and usually aims to manage the symptoms. A period of 11 years elapsed between the surgical resection of the tumor and the onset of bone metastasis observed in the present patient. This could be explained by the tumor dormancy theory, which is a poorly understood process observed in several solid neoplasms, regulated by genetic and epigenetic changes, that requires further studies to be completely comprehended.
